# Study of Rigid Cross-Linked PVC Foams with Heat Resistance

**DOI:** 10.3390/molecules171214858

**Published:** 2012-12-13

**Authors:** Aihua Shi, Guangcheng Zhang, Chenhui Zhao

**Affiliations:** School of Natural and Applied Science, Northwestern Polytechnical University, Xi’an 710129, China; E-Mails: zhangguc@nwpu.edu.cn (G.Z.); zhaohui18201@163.com (C.Z.)

**Keywords:** heat resistance, cross-linked PVC, foam plastic

## Abstract

Three heat resistant cross-linked PVC foam plastics were prepared and their performances were compared with universal cross-linked PVC structural foam. The results show that these three heat resistant foams have higher glass transition temperatures (close to 100 °C) than universal structural foam (83.2 °C). Compared with the universal structural foam, the three heat resistant foams show much higher decomposition temperature and better chemical stability due to the crosslinking of PVC macromolecular chains. The heat distortion temperature (HDT) values of the three heat resistant foam plastics are just a little higher than that of universal structural foam. The three heat resistant foam plastics have good dimensional stability at 140 °C, and when used as core material can closely adhere to the face plates in medium temperature curing processes. Compared with universal structural foam, the three heat resistant foam plastics have slightly better mechanical properties.

## 1. Introduction

Rigid cross-linked PVC foam is an ideal core material for sandwich structure composites because of its excellent features, such as outstanding stiffness and strength to weight ratios, self-extinguishing nature, good chemical resistance, sound and thermal insulation properties and low cost. Therefore, it is widely used in wind energy, marine, road and rail, aerospace, recreation and industrial applications [1].

The vacuum-assisted resin infusion (VARI) process is a very popular advanced liquid composite molding process [2], which has the outstanding advantages of low cost, being especially suitable for large size production, high performance and low porosity of molded articles and environmental friendliness [3]. So far, the VARI process has been widely used for forming ships, automobiles, aircraft, wind turbine blades and other structural parts [4].

Currently the term commercial cross-linked PVC foams generally refers to rigid polymeric foams based on polyvinyl chloride (PVC), which is modified by an interpenetrating polymer network with aromatic amides (in this paper, called universal cross-linked PVC structural foam). The PVC molecular structure in this kind of foam is linear and its glass transition temperature (T_g_) is about 80 °C [5]. This universal cross-linked PVC structural foam is fully suitable for room temperature VARI processes. In current industry practice, a medium temperature curing process (70~90 °C) is commonly used in order to improve the fluidity of the resin and the production efficiency. Heat released in the curing process under medium temperature conditions results in system temperatures of up to 120 °C, and even 140 °C. Universal cross-linked PVC structural foam core tends to shrink and release gas during the cure process in medium temperature processes, which means the core material can’t adhere to glass fiber or other faceplates. Thus research on cross-linked PVC foam plastics with higher heat resistance has significant engineering application value.

There are several methods to improve the heat resistance of a PVC resin, such as copolymerization [6], crosslinking [7], halogenation [8] and blending modifications [9]. Crosslinking occupies an important position in numerous modification methods, and plays an active role in overcoming the defects of PVC, such as low softening point and poor dimensional stability at elevated temperatures [10]. Chemical crosslinking methods of PVC mainly include peroxide crosslinking [11,12,13], silane crosslinking [14,15] and triazine compound crosslinking [16], *etc*. Most studies are focused on the crosslinking of flexible PVC foam, and seldom does research pay attention to rigid PVC foam crosslinking because of the difficulties involved. In this paper, three heat resistant cross-linked PVC foam plastics were successfully prepared and compared with universal cross-linked PVC structural foam. Two kinds of foams are prepared by the following method: trimethylolpropane trimethacrylate (TMPTMA) or triallyl isocyanurate (TAIC) serving as crosslinking agent are added into a standard universal cross-linked PVC structural foam formula. The macromolecular chains of PVC are cross-linked by crosslinking agent under the action of initiator, and thus a foam plastic with an interpenetrating polymer network structure is formed by winding with the cross-linked network generated by the action of isocyanate, anhydride and water (marked as heat resistant cross-linked PVC foam I and heat resistant cross-linked PVC foam II, respectively). The third cross-linked PVC foam plastic is prepared by the following method: copolymerization of maleic anhydride (MAH) and acrylonitrile (AN) is followed by grafting with PVC macromolecular chains, and then reacted with isocyanate and water (marked as heat resistant cross-linked PVC foam III). The three foam plastics obtained have good heat resistance, and are very compatible with higher processing or service temperatures, so they should have extensive applications and a bright development future.

**Scheme 1:**
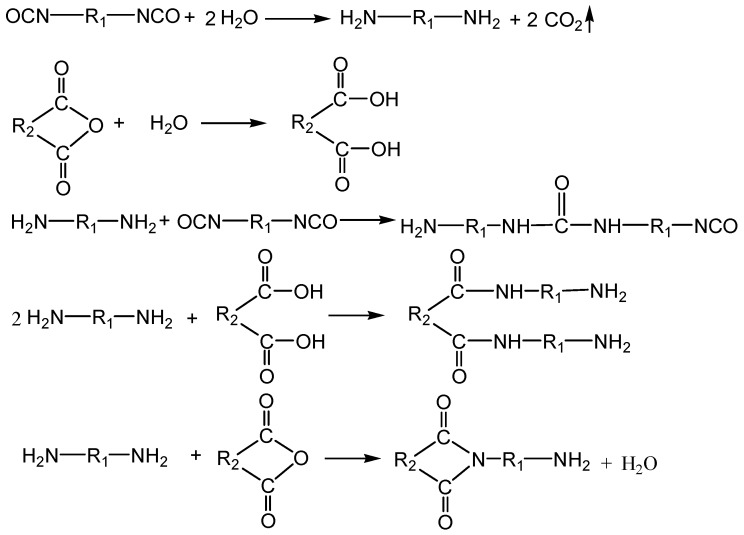
The schematic diagram of the boiled foaming reaction mechanism of universal cross-linked PVC structural foam.

**Scheme 2:**
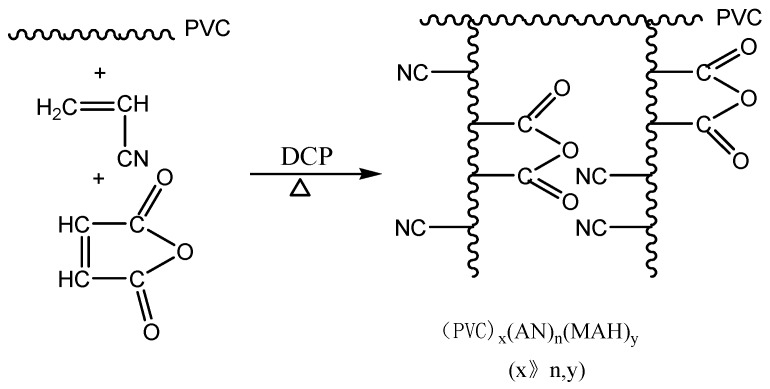
The schematic diagram of the molded process reaction mechanism of heat resistant cross-linked PVC foam III.

**Scheme 3:**
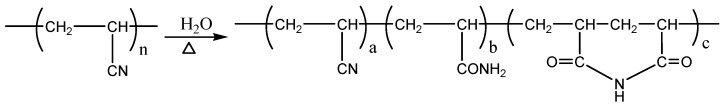
The schematic diagram of acrylonitrile hydrolysis reaction mechanism in the boiled foaming process.

The preparation of universal cross-linked PVC structural foam has been reported in previous research [1] and other literatures [17]. The reaction mechanism can be described as follows: a paste-like product was gelled in a molding process, while the gas generated by decomposition of the chemical foaming agent was dispersed in the gelation molded block, and a semi-foamed molded product was obtained after cooling. Then, in the boiled forming process, a polyurea/polyamide/polyimide crosslinking network was formed by the reaction of isocyanate, anhydride and water. This network embraced the PVC chains giving an entangled structure and a semi-interpenetrating polymer network was obtained. The decomposition mechanism of foaming agents have been reported [1,18,19], and the schematic diagram of the boiled foaming reaction mechanism is depicted in Scheme 1. Peroxide and crosslinking agent are introduced into heat resistant cross-linked PVC foam I and II. Except for the same reaction of universal cross-linked PVC structural foam in the boiled foaming process, peroxide crosslinking of PVC also exists in the molded process. There is a lot of research focused on the peroxide crosslinking of PVC to improve its heat resistance [11,12,13,20], and the crosslinking mechanism is well known, but it has not been used in the preparation of rigid low density cross-linked PVC foam plastics. Vinyl monomer and unsaturated anhydride are introduced in heat resistant cross-linked PVC foam III. Except for the similar reactions among of isocyanate, anhydride and water to universal cross-linked PVC structural foam, the graft copolymerization also exists. Scheme 2 shows the general scheme of the graft copolymerization mechanism. In addition, the nitrile group induced by the graft copolymer will be hydrolysed in the boiled foaming process, as shown in Scheme 3.

## 2. Results and Discussion

### 2.1. Gel Content Measurement

Gel content measurements were conducted to determine the degree of crosslinking of the samples. All foam plastics were controlled with the foam density of 70 kg/m^3^ for comparison, and the gel content results are shown in Table 1.

As shown in Table 1, the gel content of molded products of universal cross-linked PVC structural foam is 0, while that of foam plastics is 49.3%. This result implies that the crosslinking reaction has occurred in the boiled foaming process, but not in the molded process. The gel contents of molded products of heat resistant cross-linked PVC foam I and II are not 0, and that of foam plastics of heat resistant cross-linked PVC foam I and II are much higher than that of universal cross-linked PVC structural foam. This result implies that the degree of crosslinking is increased by the peroxide crosslinking of PVC in the molded process. The gel contents of the heat resistant cross-linked PVC foam III molded product is 0, and that of foam plastic of heat resistant cross-linked PVC foam III reaches as high as 96.4%. This result implies that a highly cross-linked heat resistant PVC foam III is realized by the graft copolymerization.

### 2.2. Thermal Mechanical Analysis (TMA)

**Figure 1:**
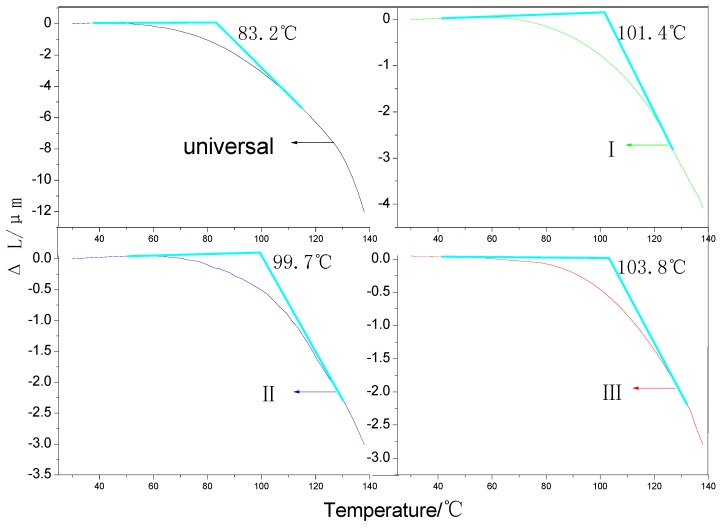
TMA curves of foam plastics.

TMA tests were carried out for the determination of T_g_, which is related to the foam density. Therefore, the foam densities were controlled the same for comparison. TMA curves of heat resistant cross-linked PVC foam I, II, III and universal cross-linked PVC structural foam with the foam density of 70 kg/m^3^ are shown in Figure 1. As shown in the figure, all the TMA curves begin to deform at 60 °C. T_g_ is obtained from the intersection of the bitangent of the curves. According to the TMA results, T_g_ values of universal cross-linked PVC structural foam and heat resistant cross-linked PVC foams I, II, III with a foam density of 70 kg/m^3^ are 83.2 °C,101.4 °C, 99.7 °C and 103.8 °C, respectively. The results show that T_g_ values of foam plastics are significantly increased and heat resistant performance improves after the crosslinking of the PVC chains. In other words, the T_g_ values obtained by TMA as the upper limit of continuous operating temperature is enhanced due to the crosslinking of PVC chains.

### 2.3. Differential Scanning Calorimetry (DSC) Analysis

**Figure 2:**
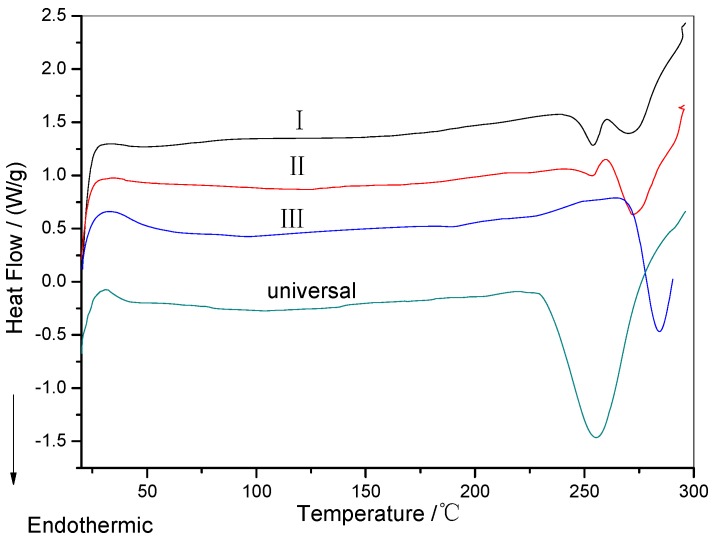
DSC curves of foam plastics.

Figure 2 shows the DSC analysis results for heat resistant cross-linked PVC foams I, II, III and universal cross-linked PVC structural foam.

As shown in Figure 2, the peak temperature of the endothermic area caused by PVC decomposition in the universal cross-linked PVC structural foam is 259 °C. According to the DSC curves of heat resistant cross-linked PVC foam I and II, the endothermic peak caused by PVC decomposition is decomposed into two peaks. This can be explained by that two forms of PVC exist in heat resistant cross-linked PVC foams I and II, one of which involves linear PVC penetration in the cross-linked network, while the other is cross-linked PVC. The peak temperature of the endothermic area caused by PVC decomposition in heat resistant cross-linked PVC foam III is 284 °C, which is related to the almost completely cross-linked structure. This matches well with the former gel content measurements and TMA tests.

### 2.4. Thermogravimetric Analysis (TGA) and Derivative Thermogravimetry (DTG) Analysis

**Figure 3:**
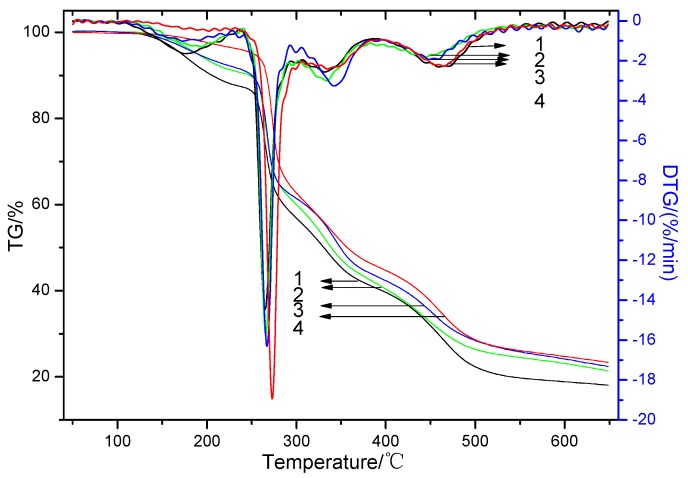
TGA/DTG curves of foam plastics (**1**) universal structural foam, (**2**) heat resistant foam I, (**3**) heat resistant foam II, (**4**) heat resistant foam III.

Figure 3 demonstrates the TG/DTG analysis for heat resistant cross-linked PVC foams I, II, III and universal cross-linked PVC structural foam. The parameters of 5% weight loss temperature (T_d_^5^), 10% weight loss temperature (T_d_^10^), rapid decomposition temperature of PVC (T_d_), 50% weight loss temperature (T_d_^50^) and residual weight are reported in Table 2.

It is obvious in Figure 3 and Table 2 that all of the three kind heat resistant cross-linked PVC foams have higher T_d_^5^, T_d_^10^, T_d_, T_d_^50^ and residual weight than the universal cross-linked PVC structural foam. The decomposition temperature and weight loss temperature of heat resistant cross-linked PVC foam III improved significantly, while heat resistant cross-linked PVC foam I and II just improve slightly. This phenomenon indicates that the crosslinking of PVC chains is beneficial to heat resistance performance. The descending order of heat stability of the four kinds of foams is summarized as follows: heat resistant cross-linked PVC foam III, heat resistant cross-linked PVC foam II, heat resistant cross-linked PVC foam I, universal cross-linked PVC structural foam.

### 2.5. Heat Distortion Temperature (HDT) Analysis

**Figure 4:**
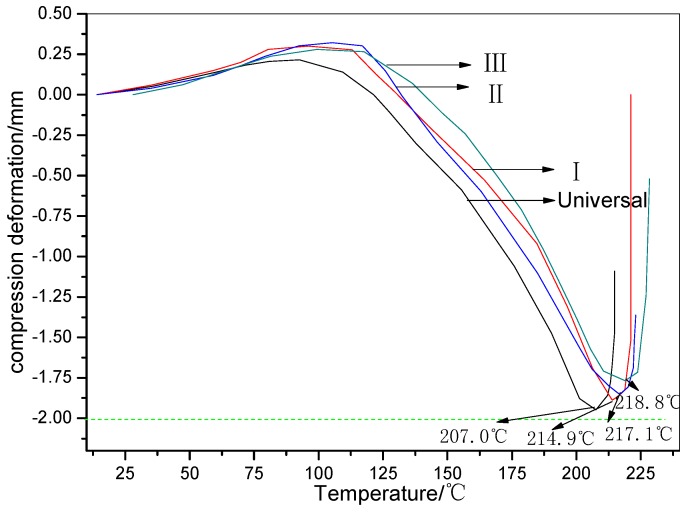
Heat distortion temperature curves of foam plastics.

The HDT of foam plastic increases with elevated foam density. Therefore, the density was kept at 70 kg/m^3^ for all four foams for comparison. Figure 4 shows the corresponding HDT curves.

As shown in Figure 4, the thickness of universal cross-linked PVC structural foam increased from room temperature and then decreased after 95 °C. However, the thicknesses of heat resistant cross-linked PVC foams I, II, III are all increased until the temperature reaches 120 °C, which is about 25 °C higher than in universal cross-linked PVC structural foam. This phenomenon is related to the higher T_g_ values of these three kinds heat resistant cross-linked PVC foams than that of universal cross-linked PVC structural foam. The heat expansion degree of gas in cells is so large that the thickness increases significantly as the temperature increases. Foam plastics reach a high elastic state when the temperature exceeds T_g_, and foam plastics are compressed caused by gas escape through the cell walls. When the temperature is high enough (exceeding 200 °C), the matrix resin of cell walls and cell struts undergo further crosslinking curing, which means the gas cannot escape from the cells and this causes a secondary expansion.

According to the DIN53424 standard, for the rigid, closed-cell foam plastics the temperature when the compression deformation reaches to 2 mm is defined as HDT. However, in this work, the thickness decrease under compressive load cannot reach 2 mm, so the temperature when the thickness of foam decreases to the lowest point is considered as HDT. As shown in Figure 4, the HDT values of heat resistant cross-linked PVC foams I, II, III and universal cross-linked PVC structural foam are 214.9 °C, 217.1 °C, 218.8 °C and 207.0 °C, respectively. The HDT values obtained by this method far exceed the T_g_ of cross-linked PVC foam plastic, showing that they have no engineering application value. However, the research of the deformation behavior in the heating process can provide theoretical basis for improving the heat resistant performance.

### 2.6. Dimension Stability Analysis

The test for dimension stability of foam plastics with foam density of 70 kg/m^3^ was implemented according to ISO 2796. At 160 °C, brittle rupture will take place when foam plastics expand to a certain extent. The heating time (at 160 °C) for heat resistant cross-linked PVC foams I, II, III and universal cross-linked PVC structural foam to begin rupturing are 12 h, 14 h, 16 h and17 h, respectively. Therefore, the data of weight and volume change rate at the point of 1h before the rupture is selected. Table 3 lists the dimension stability values of the different foam plastics.

As shown in Table 3, the dimensional stability rule of heat resistant cross-linked PVC foam under high temperature conditions is consistent with that of universal cross-linked PVC structural foam. Volume shrinkage occurs in all foams at temperatures below 120 °C, and then the shrinkage degree decreased with elevating temperature. Brittle rupture will take place in the foam plastics after excessive expansion at 160 °C. When the temperature exceeds the T_g_ of the foam plastics, the weight loss rate and volume shrinkage rate of universal cross-linked PVC structural foam increase rapidly, while they are small for the three kinds of heat resistant cross-linked PVC foam under the same temperature conditions. It was also found that the weight and volume change rate of the three heat resistant cross-linked PVC foam increase slightly more than that of universal cross-linked PVC structural foam with elevating temperature. This phenomenon may be because that those three kinds heat resistant cross-linked PVC foam have higher T_g_ values, and the chains in the cell wall and cell struts are difficult to move below 120 °C, so the foam shrinkage is avoided. Further curing may take place in matrix resin of cell walls and cell struts when the temperature is higher than 140 °C, thus the volume change rate of the three kinds of heat resistant cross-linked PVC foam is small when the heating temperature is below 140 °C. Furthermore, the heating time before brittle rupture of these three kinds of elevated temperature PVC structural foam is longer than that of universal cross-linked PVC structural foam. This is because the degradation temperature of the PVC resin in the heat resistant cross-linked PVC foams is elevated.

### 2.7. Mechanical Properties Analysis

Table 4 demonstrates the mechanical properties of cross-linked PVC foam plastics with a density of 70 kg/m^3^.

As shown in Table 4, the mechanical properties of heat resistant cross-linked PVC foam I and II are slightly higher than universal cross-linked PVC structural foam. This may be because all these foam plastics have interpenetrating polymer network structures. Under the condition of similar cell structure, the mechanical properties are decided by the performance of the matrix resin. Although the crosslinking of PVC chains is conducive to improving the strength of the matrix resin, the strength of the polyurea/polyamide/polyimide network is not changed. In addition, only slight improvement is observed in heat resistant cross-linked PVC foams I and II. Compressive strength and modulus, tensile strength and modulus and shear strength of heat resistant cross-linked PVC foam III are slightly higher than that of universal cross-linked PVC structural foam. However, the elongation at break in the tensile and shear strength testing is reduced. This may be because the PVC chains in heat resistant cross-linked PVC foam III are cross-linked by a copolymer of MAH and AN, and the high degree of crosslinking leads to less flexibility and less extension.

## 3. Experimental

### 3.1. Materials

Polyvinyl chloride paste resin (PVC) is purchased from VESTOLIT GmbH & Co KG, Marl, Germany. Liquified methylene bis-phenyl diisocyanate (MDI-L) is purchased from Bayer AG, Leverkusen, Germany. Methylhexahydrophthalic anhydride (MHHPA) is purchased from Puyang Huicheng Chemical Co., Ltd, Puyang, China. Azodicarbonamide (AC) is purchased from Kaifeng Dongda Chemical Co., Ltd, Kaifeng, China. Azodiisobutyronitrile (AIBN) is purchased from Shanghai Sanpu Chemical Co., Ltd, Shanghai, China. Epoxidized soybean oil (ESO) is purchased from Zibo Kailian Chemical Co., Ltd, Zibo, China. Dicumyl peroxide (DCP) is purchased from Sinopharm Chemical Reagent Co., Ltd, Xi’an, China. Trimethylolpropane trimethacrylate (TMPTMA)/triallyl isocyanurate (TAIC) is purchased from Shanghai Farida chemical Co., Ltd., Shanghai, China. Maleic anhydride (MAH) is purchased from Tianjin Fuchen Chemical Co., Ltd, Tianjin, China. Acrylonitrile (AN) is purchased from Xi’an Organic Chemical Co., Ltd, Xi’an, China. All solid reagents were dried before used.

### 3.2. Experiment Preparation

Table 5 gives the formulas of three heat resistant cross-linked PVC foams and universal cross-linked PVC structural foam. Foam plastics are prepared as follows: firstly, all materials are blended until a perfectly homogeneous paste-like product is formed. Secondly, the paste-like product is molded for 20 min under the conditions of 170 °C and 15 MPa. Thirdly, the molded product is exposed to a steam of hot water (97 °C) to expand until the desired density is obtained. Finally, foam plastics are treated in warm water (50 °C) to remove any residual active ingredients.

### 3.3. Characterization

#### 3.3.1. Gel Content Measurement

The degree of crosslinking can be estimated through the gel fraction. The gel contents of molded products and foam plastics were determined gravimetrically by Soxhlet extraction for 24 h using tetrahydrofuran (THF) as the solvent. Approximately 0.5 g of the molded product or foam plastic (m_0_) was cut into small pieces. After the extraction cycle, the sample was dried to constant weight (m_1_). Taking into account the initial insoluble fraction (f) of the sample, the gel content (GC) can be calculated according to expression (1):
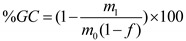
(1)

#### 3.3.2. Heat Resistance Analysis

The glass transition temperature (T_g_) of foam plastic was obtained using an Mettler-Toledo TMA 840 thermomechanical analyzer. The temperature was risen from 30 °C to 140 °C at the speed of 5 °C/min, and the sample size for TMA analysis was 10 mm × 8 mm × 6 mm. Heat transformation of foam plastic was recorded by a TA Q2910 differential scan calorimeter. The temperature was increasedfrom 25 °C to 300 °C at a rate of 10 °C/min. Weight change and thermal decomposition of foam plastic were analyzed by a NETZSCH 209 F1 thermogravimetric analyzer from 50 °C to 650 °C at the rate of 10 °C/min. The atmosphere used was nitrogen with a flow rate of 30 mL/min. HDT of foam plastic was measured by a Martin heating test box 110A which was produced by the instrument repair center of Northwestern Polytechnical University. Dimensional stability of foam plastic was measured in a 101-2A drying oven under forced convection. The dimensional stability test is implemented according to ISO 2796.

#### 3.3.3. Mechanical Property Analysis

Mechanical properties of foam plastics were measured by an MTS CMT7204 universal testing machine. Flatwise compressive properties, tensile properties, shear properties of foam plastics were tested according to standards ASTM D1621, D638, C273, respectively. At least five specimens were tested for each sample.

## 4. Conclusions

In this work, three heat resistant foam plastics are prepared and their performances are compared with universal cross-linked PVC structural foam. The T_g_ values of heat resistant cross-linked PVC foams I, II, III are 101.4 °C, 99.7 °C and 103.8 °C, respectively, which are higher than that of the universal structural foam. The crosslinking of PVC macromolecular chains makes heat resistant cross-linked PVC foams I, II, III have higher decomposition temperatures and better heat stability than universal structural foam. HDT values of heat resistant cross-linked PVC foams I, II, III are just a little higher than that of universal cross-linked PVC structural foam. The three heat resistant foam plastics all have good dimensional stability and small volume change rates. The volume change rates of the three heat resistant foam plastics are just less than half of that of the universal structural foam at 140 °C. Compared with universal cross-linked PVC structural foam, these three heat resistant cross-linked PVC foam plastics have slightly better mechanical properties.

## Figures and Tables

**Table 1:** Gel contents of molded products and foam plastics.

Foam types	Gel content/%	
	Molded products	Foam plastics
universal cross-linked PVC structural foam	0	49.3
heat resistant cross-linked PVC foam I	30.6	80.1
heat resistant cross-linked PVC foam II	35.5	86.3
heat resistant cross-linked PVC foam III	0	96.4

**Table 2:** TGA values of foam plastics.

Foam type	T_d_^5^/°C	T_d_^10^/°C	T_d_/°C	T_d_^50^/°C	Residual weight/%
universal cross-linked PVC structural foam	172	206	257	331	18.08
heat resistant cross-linked PVC foam I	189	245	259	339	21.41
heat resistant cross-linked PVC foam II	189	250	259	349	22.38
heat resistant cross-linked PVC foam III	252	267	265	357	23.38

**Table 3:** Dimension stability of foam plastics.

Temperature		80 °C	100 °C	120 °C	140 °C	160 °C ^a^
Weight change rate/%	heat resistant foam I	−0.95	−1.73	−2.36	−4.81	−7.20
	heat resistant foam II	−0.93	−1.73	−2.31	−4.73	−7.04
	heat resistant foam III	−0.85	−1.58	−2.24	−3.98	−6.33
	universal structural foam	−1.06	−2.48	−3.89	−5.17	−10.97
Volume change rate/%	heat resistant foam I	−2.85	−4.48	−5.74	−2.03	+28.41
	heat resistant foam II	−2.73	−4.37	−5.39	−1.04	+32.27
	heat resistant foam III	−2.03	−3.52	−5.04	+1.16	+34.06
	universal structural foam	−3.41	−8.51	−11.23	−5.77	+15.64

^a^ Test values at 160 °C are obtained for 1h before the rupture, while the data at other temperatures are obtained at the end of the measurement (after heating for 48 h).

**Table 4:** Mechanical properties of foam plastics.

Foam plastic	I	II	III	Universal
Compressive strength /MPa	1.17	1.23	1.21	1.11
Compressive modulus/Mpa	51.5	52.9	52.1	49.4
Tensile strength /Mpa	1.77	1.76	1.73	1.69
Tensile modulus /Mpa	57.3	58.2	57.0	55.8
Elongation at break /%	9.1	9.0	5.5	8.4
Shear strength /Mpa	1.01	0.99	0.92	0.95
Shear modulus /Mpa	29.8	30.2	29.8	28.4

**Table 5:** Formulas of cross-linked PVC foam plastics.

Formulation	Universal	I	II	III
PVC	100	100	100	100
MDI-L	64	64	64	64
MHHPA	29	29	29	-
AIBN	3	3	3	3
AC	1	1	1	1
ESO	3	3	3	3
DCP	-	1	1	1
TMPTMA	-	5	-	-
TAIC	-	-	10	-
MAH	-	-	-	17
AN	-	-	-	4
